# Resilience and Social Support as Predictors of Life Satisfaction in Healthy Older Adults: A Moderation Analysis

**DOI:** 10.62641/aep.v54i3.2218

**Published:** 2026-06-15

**Authors:** Lorena González-García, Aranzazu Duque, Mireia Molins, Marta Aliño, Francisco Molins, Patricia Mesa-Gresa

**Affiliations:** ^1^Department of Social Psychology, Faculty of Psychology and Speech Therapy, Universitat de València, 46010 Valencia, Spain; ^2^Psychology and Quality of Life Research Group, Faculty of Health Sciences, Universidad Internacional de Valencia, 46002 Valencia, Spain; ^3^Department of Psychobiology, Faculty of Psychology and Speech Therapy, Universitat de València, 46010 Valencia, Spain

**Keywords:** resilience, social support, life satisfaction, older adults, aging

## Abstract

**Background:**

Previous literature has highlighted the importance of psychosocial factors such as the perception of an adequate social support network or the subjective experience of life satisfaction in promoting well-being in older adults and facilitating healthy aging processes. The objective of the present study was to analyze the possible relationship between the level of resilience and social support in healthy older adults, and how these variables may influence experiences of life satisfaction. Furthermore, potential moderation relationships between these variables were analyzed.

**Methods:**

The sample consisted of 42 healthy older adults (71.4% women), with a mean age of 66.57 years (standard deviation (SD) = 5.82). Participants completed a battery of questionnaires that included a sociodemographic questionnaire, the Connor-Davidson Resilience Scale (CD-RISC), the Duke-UNC Functional Social Support Questionnaire (Duke-UNC-11), and the Satisfaction With Life Scale (SWLS).

**Results:**

The results of the multiple regression analyses indicate that resilience and social support positively and significantly predict life satisfaction. Furthermore, moderation analyses indicate that social support moderates the relationship between resilience and life satisfaction, jointly explaining 44.98% of the variance of this indicator of well-being. Specifically, the positive association between resilience and life satisfaction was stronger at lower levels of perceived social support.

**Conclusions:**

These findings suggest that resilience may play a particularly relevant role in life satisfaction when perceived social support is limited. However, the small sample size, the predominance of women, and the selective recruitment of healthy older adults warrant caution when generalizing the results. Overall, the findings support the need to expand the study of the correlations of well-being in old age, as well as to develop intervention programs aimed at promoting optimal aging that include strategies to improve resilience and foster functional social support networks.

## Introduction

Population aging represents a complex demographic phenomenon of great relevance in the 21st century and a global challenge, with significant social, health, economic, and population-related consequences [1, 2]. To address the challenges resulting from the rapid aging of the population, it is essential to investigate which factors may contribute to the promotion of healthy aging, enabling older adults to enjoy adequate cognitive functioning as well as greater well-being and quality of life [3]. In this regard, scientific literature highlights the importance of studying how various psychosocial factors—such as social support, personality, or health status—can influence well-being and life satisfaction during this stage of life [4, 5, 6].

Gaining a better understanding of the situation of older adults requires recognizing the significant and frequent transformations experienced during this stage of life, including changes in health status, retirement, the loss of significant others, or changes in financial situation. All these changes can lead to a deterioration of emotional well-being, contributing to sleep disturbances, dysregulation of stress response, and disorders such as anxiety and depression, which in turn worsen quality of life and cognitive performance, and have been associated with an increased risk of dementia [7]. Furthermore, during the later years of life, people often experience increased isolation and loneliness, which are associated with greater sedentarism, fewer health-promoting behaviors, and a higher prevalence of mood disorders such as anxiety or depression [8].

The changes described above may lead to a decline in subjective well-being during this stage of life. Among the most studied indicators of subjective well-being in the older population, life satisfaction stands out [9], defined as the global and subjective cognitive evaluation of the extent to which an individual is satisfied with their life based on their goals and achievements [10]. The concept of life satisfaction involves assessing and comparing actual life circumstances to expected ones [11]. When considering the evolution of life satisfaction across the lifespan, it has often been assumed that it may decline in older age due to increased dependency, health problems and the loss of close relationships, although empirical findings suggest more complex and non-linear patterns [12]. However, previous literature has argued that life satisfaction and happiness increase with age [13]. Factors influencing life satisfaction in older adults include health status, economic level, social support, type of pension, and intergenerational support [9]. In this line, previous research has shown that higher life satisfaction is associated with a lower risk of chronic illness and mortality, as well as with more favorable aging outcomes [14, 15]. Taken together, this evidence highlights the importance of examining the factors that contribute to life satisfaction in older adults and how they interact.

One of the personality traits most frequently associated with healthy aging and subjective well-being in older adults is resilience [16], understood as the ability to quickly and effectively cope with and recover from difficulties, stress, and adversity through adaptation [17, 18]. Positive psychology emphasizes the importance of resilience in promoting mental health adaptations [19]. It is suggested that in adverse situations, highly resilient individuals are expected to adapt to and recover from setbacks and difficult experiences more easily [20, 21]. Studies focused on older adults have confirmed that those with higher levels of resilience appear to have greater capacity to thrive in the face of adversity or disruptive events [22, 23]. Research on the relationship between resilience and life satisfaction has shown that increased resilience predicts greater life satisfaction [24, 25]. In fact, resilience is positively and significantly correlated with higher life satisfaction throughout the lifespan [26, 27]. Resilient older adults are considered to have greater flexibility, higher confidence, longer life expectancy, more capacity to forgive others, a greater sense of purpose, more social participation and a more positive outlook on life and the future [28]. However, although resilience is a key individual factor in promoting life satisfaction, its effects may be influenced by contextual factors. In this sense, social support has been identified as a relevant external resource that contributes both to the maintenance of resilience and to the promotion of well-being in older adults [29, 30].

Focusing on the role that social support plays in promoting well-being in old age, experts have long recognized the importance of this contextual factor in the prediction of life satisfaction in older adults [31, 32]. According to Cohen and McKay [33], social support includes the psychological and physical resources provided by social networks that help individuals cope with stress and negative moods. Social support has been shown to be associated with improvements in mental health [34], and people who receive support from family, friends, or professionals tend to report higher levels of happiness and life satisfaction [35] and higher resilience [29]. Along these lines, Zhou* et al*. [36] propose that social support intervenes in the relationship between resilience and life satisfaction. Thus, given the importance of this contextual factor, intervention programs have been developed to foster social support networks and consequently strengthen resilience and well-being in older individuals [37, 38].

The findings to date underscore the relevance of personal factors, such as resilience, and social factors, such as perceived social support, for maintaining adequate mental health and improving experiences of life satisfaction and other well-being indicators. However, there is still limited literature that jointly examines the role of both resilience and social support in predicting life satisfaction among older adults. Moreover, previous research has reported inconsistent findings regarding the interplay between resilience and social support, particularly in relation to whether these variables operate through mediation or moderation mechanisms. From a mediation perspective, some studies suggest that one variable may act as an explanatory pathway through which the other influences well-being outcomes. For instance, Zheng* et al*. [26] found that older adults’ resilience partially mediates the relationship between perceived filial support and life satisfaction. Similarly, research in other populations has shown that perceived social support may mediate the relationship between resilience and quality of life in women with breast cancer [36], as well as between resilience and burnout in caregivers of older adults [39].

In contrast, other theoretical and empirical approaches propose that resilience and social support may function as interacting resources, such that the effect of one depends on the level of the other [33]. From this perspective, moderation models assume that psychosocial resources do not operate independently but rather in a context-dependent and potentially compensatory manner. For example, social support may buffer the effects of lower individual resilience, consistent with the buffering hypothesis, whereas resilience may become particularly relevant in contexts where social support is limited, reflecting compensatory and interactive processes among psychosocial resources [40, 41]. To date, there is a relative scarcity of studies explicitly testing moderation models in older adult populations, and the conditions under which resilience and social support interact to influence life satisfaction remain insufficiently understood and, therefore, further research is needed to clarify their interaction.

The state of the art, as well as the importance of life satisfaction in promoting healthy aging, highlights the need for further exploration of the role of resilience and perceived social support in predicting well-being. Although previous studies have examined these variables independently, and some have explored their interplay through mediation models [26, 36], there is still limited evidence regarding how resilience and social support jointly operate in older adults, particularly from an interactional (moderation) perspective. This gap is especially relevant in the context of aging, as older adults are more likely to experience significant life changes (e.g., retirement, health-related challenges, social losses) that may alter both the availability of external resources (e.g., social support) and the reliance on internal coping capacities (e.g., resilience) [3]. In this sense, it is plausible that the impact of resilience on life satisfaction is not uniform but rather depends on the level of available social support, reflecting a context-dependent process. Furthermore, most previous studies examining the interplay between resilience and social support have been conducted in clinical or specific populations (e.g., patients or caregivers) [25, 36], while less attention has been paid to healthy, community-dwelling older adults, whose psychosocial functioning may differ substantially. Understanding these dynamics in this population is essential for identifying protective factors that contribute to successful and satisfying aging [16].

Thus, the general aim of the present study was to analyze the relationship between resilience, perceived social support, and life satisfaction in healthy older adults. To this end, the first specific objective was to determine the predictive value of resilience and social support on life satisfaction. The second specific objective was to analyze whether social support moderates the relationship between resilience and life satisfaction among older adults. Results obtained through this work may help to clarify the relationship between the studied variables and may serve as a foundation for designing interventions aimed at strengthening psychosocial resources in older adults, thereby promoting healthier and more satisfying aging.

Based on the aforementioned specific objectives and current literature, the following hypotheses were proposed: (H1) Resilience and perceived social support will positively predict life satisfaction [25, 31]; and (H2) Social support will moderate the relationship between resilience and life satisfaction in older adults [29, 33, 36].

## Materials and Methods

### Participants

The study participants were 42 older adults, aged between 57 and 83 years (mean age = 66.57; standard deviation (SD) = 5.82), with 71.4% of the sample being women. Participants were recruited using convenience sampling conducted within a university program for older adults and provided informed consent prior to the study. Given the exploratory nature of the study and the restricted number of eligible volunteers available during the recruitment period, the final sample size was determined by the number of participants who met the inclusion criteria and agreed to participate. The inclusion criteria were: (a) being 55 years of age or older; and (b) scoring above the cutoff point on the Mini-Mental State Examination (MMSE ≥ 24), indicating the absence of significant cognitive impairment [42]. The exclusion criteria included the presence of sensory or motor impairments that could interfere with the completion of the questionnaires. Thus, the participants of our study were considered healthy older adults insofar as they were community-dwelling individuals, actively engaged in educational activities, without evident cognitive impairment, and functionally able to complete the assessment instruments. This study was conducted in accordance with the Declaration of Helsinki and was approved in February 2024 by the Ethics Committee of the Universitat de València where the study was conducted (Reference: 2023-PSILOG-2558999).

### Instruments

Sociodemographic Questionnaire. It was developed “ad hoc” for this study. The information collected includes variables such as gender, age, educational level, marital status, number of children, and number of people living with.

Mini-Mental State Examination Questionnaire [43]. The Spanish adaptation [42] of this questionnaire is extensively used in clinical and research settings to measure cognitive impairment, including simple tasks in a number of areas: the test of time and place, the repeating lists of words, arithmetic such as serial subtractions of seven, language use and comprehension, and basic motor skills. It consists of 30 items, with a maximum score of 30 points; lower scores indicate greater cognitive impairment. The study validation reported a sensitivity of 0.85, a specificity of 0.90, and an intra-rater reliability of 0.93 [42]. In the present sample, Cronbach’s alpha was 0.74.

Connor-Davidson Resilience Scale (CD-RISC) [44]. The Spanish adaptation [45] of this scale assesses resilient behaviors in adults. It consists of 25 items answered using a five-point Likert scale ranging from 0 “not at all” to 4 “almost always”, yielding a total score from 0 to 100, with higher scores indicating greater resilience. An example item is “I am able to adapt when changes occur”. This scale has been used in other studies, showing a reliability of α = 0.87 [46]. In the present sample, Cronbach’s alpha was 0.90.

Duke-UNC Functional Social Support Questionnaire (Duke-UNC-11) [47]. The Spanish adaptation [48] of this instrument assesses perceived functional social support through 11 items grouped into two dimensions: confidential support (the ability to communicate with others) and affective support (demonstrations of love, affection, and empathy). An example of an item for the confidential support dimension is “I have the opportunity to talk to someone about my personal and family problems”, and an example of an item for the affective support dimension is “I receive love and affection”. Items are answered using a five-point Likert scale ranging from 1 “Much less than you would like” to 5 “As much as you would like”. Scores range from 11 to 55 points, with higher scores indicating greater perceived social support. In the Spanish validation, a score of 32 or lower indicates a low level of perceived social support, while a score above 32 indicates a normal level of perceived social support. Previous studies have demonstrated adequate internal consistency of the instrument (global evaluation: α = 0.89; confidential support dimension: α = 0.87; affective support dimension: α = 0.74) [49]. In the present sample, Cronbach’s alpha was 0.88.

Satisfaction With Life Scale (SWLS) [10]. The Spanish adaptation [50] of this scale is used to assess psychological well-being and quality of life by analyzing global cognitive judgments regarding the life of the person being evaluated. It is composed of 5 items answered using a seven-point Likert scale ranging from 1 “Strongly disagree” to 7 “Strongly agree”, yielding a total score ranging from 5 to 35, with higher scores indicating greater life satisfaction. An example item is “In most aspects my life is the way I want it to be”. Previous studies have shown good internal consistency of the instrument (α = 0.86) [51]. In the present sample, Cronbach’s alpha was 0.85.

### Procedure

Samples were collected between February 2024 and May 2024. The study was conducted in two phases: one carried out in the laboratory and the other in which participants completed questionnaires on their own. Specifically, the laboratory sessions took place from Monday to Thursday, between 9:00 AM and 12:45 PM, and lasted approximately 45 minutes to an hour. Participants were contacted and arranged to meet at a designated location at the faculty where the study was conducted. Once in the laboratory, they were explained the general procedure, and they read and signed the corresponding informed consent form. Participation was voluntary, and no financial or other incentives were provided. All participants received a brief assessment of their cognitive status using the MMSE questionnaire. They were then provided with the full set of questionnaires, which they completed at home in order to ensure a comfortable and unhurried response process. Upon returning the completed questionnaires to the laboratory, participants had the opportunity to review their responses with a researcher and clarify any doubts before final submission.

### Analysis

Outliers were identified using the 2.5 standard deviation method. Assumptions were evaluated using a combination of numerical and graphical procedures. Distributional properties were examined through normality tests and visual inspection of Q-Q plots, while bivariate scatterplots were inspected to assess the plausibility of linear relationships and detect potential extreme cases. For regression analyses, residual diagnostics were also examined, given that the normality assumption in linear models pertains to the residuals rather than to the raw observed variables. Overall, no severe departures from linearity or other major assumption violations were observed, and regression residuals were consistent with approximate normality. Therefore, the use of parametric analyses was considered appropriate.

Given the exploratory nature of the study, a sensitivity analysis was conducted to estimate the magnitude of effects that could be detected with the available sample size. With N = 42 and α = 0.05, the study had approximately 80% power to detect correlations in the range of *r* ≈ 0.41–0.42 and regression effects of moderate magnitude.

Exploratory analyses were then conducted to investigate the distribution of variables and their interrelationships. Subsequently, regression analyses were performed to determine whether resilience and social support could predict life satisfaction and to evaluate which of the two variables had greater predictive value. For interpretive purposes, both unstandardized coefficients (B) and standardized coefficients (β) were examined, and collinearity diagnostics (tolerance and variance inflation factor (VIF)) were inspected for the multiple regression model.

Finally, moderation analyses were conducted to further explore the dynamic between resilience and life satisfaction, incorporating social support as a moderator, and using Hayes’ PROCESS macro for SPSS (Model 1), estimated through ordinary least squares regression with 5000 bootstrap samples and 95% confidence intervals. The Johnson-Neyman procedure was then applied to examine these interactions in greater depth and to identify the range of values of perceived social support for which the conditional effect of resilience on life satisfaction was statistically significant. Variables were entered in their original observed metric and were not standardized prior to analysis; therefore, the Johnson-Neyman threshold is expressed in the raw score units of the perceived social support measure. The significance level (α) was set at 0.05, and partial eta squared (η^2^p) was used to indicate effect size. All analyses were conducted using IBM SPSS Statistics (version 25.0, IBM corporation, Armonk, NY, USA).

## Results

### Descriptive Statistics

The general descriptive statistics for the sample are presented in Table 1. These variables were compared by gender to assess potential differences between men and women. As shown in Table 1, no significant differences were observed in any of the measured variables. Although both resilience (*p* = 0.094) and life satisfaction scores (*p* = 0.059) indicated a trend toward significance, with men reporting slightly higher resilience and satisfaction than women, this did not reach statistical significance. Regarding the sociodemographic profile of the sample, half of the participants were married (50.0%), while the remainder were single (11.9%), in a relationship (7.1%), divorced (21.4%), or widowed (9.5%). It is important to consider the disproportionate gender distribution in the sample (71.43% women and 28.57% men), which necessitates caution when interpreting these results, as this imbalance could increase the likelihood of a Type II error.

**Table 1.** **Descriptive statistics of the scores obtained in the CD-RISC (resilience), Duke-UNC-11 (social support) and SWLS (satisfaction with life) questionnaires & gender comparison**.

Variables	Global (N = 42)	Men (N = 12)	Women (N = 30)	F	df between	df within	*p*-value	η^2^p
Age	66.57 ± 5.82	66.33 ± 5.17	66.67 ± 6.14	0.02	1	40	0.860	0.001
Resilience	73.24 ± 13.67	78.83 ± 9.39	71.00 ± 14.58	2.94	1	40	0.094^†^	0.069
Social support	44.12 ± 7.69	43.92 ± 9.02	44.20 ± 7.27	0.11	1	40	0.916	0.000
Life satisfaction	27.31 ± 5.32	29.75 ± 3.64	26.33 ± 5.62	3.76	1	40	0.059^†^	0.086

Note: Mean ± standard deviation. Men and women were contrasted through Analysis of Variance (ANOVA). CD-RISC, Connor-Davidson Resilience Scale. Duke-UNC-11, Duke-UNC Functional Social Support Questionnaire; SWLS, Satisfaction With Life Scale. A trend toward significance is indicated by ^†^ (*p* < 0.10).

### Interrelation Between Resilience, Social Support & Life Satisfaction

The analysis of Pearson correlations among the key variables indicated that resilience was positively correlated with life satisfaction, *r*(40) = 0.409, *p* = 0.007, 95% confidence interval (CI) (0.120, 0.634), suggesting that higher levels of resilience are associated with greater life satisfaction. Similarly, social support was also positively correlated with life satisfaction, *r*(40) = 0.421, *p* = 0.006, 95% CI (0.134, 0.643), indicating that higher social support is linked to greater life satisfaction. However, there was no significant correlation between resilience and social support, *r*(40) = 0.108, *p* = 0.497, 95% CI (-0.203, 0.399). These findings suggest that while both resilience and social support individually relate to life satisfaction, they do not significantly correlate with each other within this sample.

### Regression Analyses

To determine whether resilience and social support could predict life satisfaction, a multiple linear regression analysis was conducted. The predictors included in the model were total scores of resilience and social support, with life satisfaction as the dependent variable. The overall regression model was significant, F(2, 39) = 8.805, *p* = 0.001, explaining 31.1% of the variance in life satisfaction (R^2^ = 0.311, with an adjusted R^2^ = 0.27). In line with the correlation analyses, social support emerged as a significant positive predictor of life satisfaction (B = 0.264, standard error (SE) = 0.092, β = 0.381, *t* = 2.851, *p* = 0.007), indicating that higher levels of social support are associated with greater life satisfaction. Similarly, resilience was also a significant predictor (B = 0.143, SE = 0.052, β = 0.368, *t* = 2.754, *p *= 0.009), suggesting that higher resilience contributes to increased life satisfaction. These results affirm that both social support and resilience are important factors in predicting life satisfaction during aging. The standardized coefficients indicate that both predictors made a very similar contribution to the model, with a slightly stronger relative effect for social support. Moreover, collinearity diagnostics showed no evidence of multicollinearity (tolerance = 0.988 and VIF = 1.012 for both predictors).

### Moderation Analyses

Building on the insights from the previous regression analyses, a moderation analysis was performed to further explore the dynamic between resilience and life satisfaction, incorporating social support as a moderator. This analysis aimed to dissect the nuances not captured by the initial regression model, which already indicated significant roles for both resilience and social support in predicting life satisfaction.

The comprehensive model confirmed the significant predictive power of these variables, explaining approximately 44.98% of the variance in life satisfaction (F(3, 38) = 10.354, *p* < 0.001). Resilience continued to show a positive effect on life satisfaction (B = 0.765, SE = 0.206, *t *= 3.708, *p* = 0.001), a finding consistent with earlier results. Social support also maintained its significant positive impact (B = 1.314, SE = 0.350, *t *= 3.759, *p* < 0.001). However, the interaction between resilience and social support introduced a new layer of complexity (B = -0.014, SE = 0.005, *t* = -3.095, *p* = 0.004), indicating a moderating effect. This interaction significantly altered the relationship between resilience and life satisfaction, contributing an additional 13.87% to the explained variance (R^2^ change = 0.139, F(1, 38) = 9.580, *p* = 0.004).

**Fig. 1.**
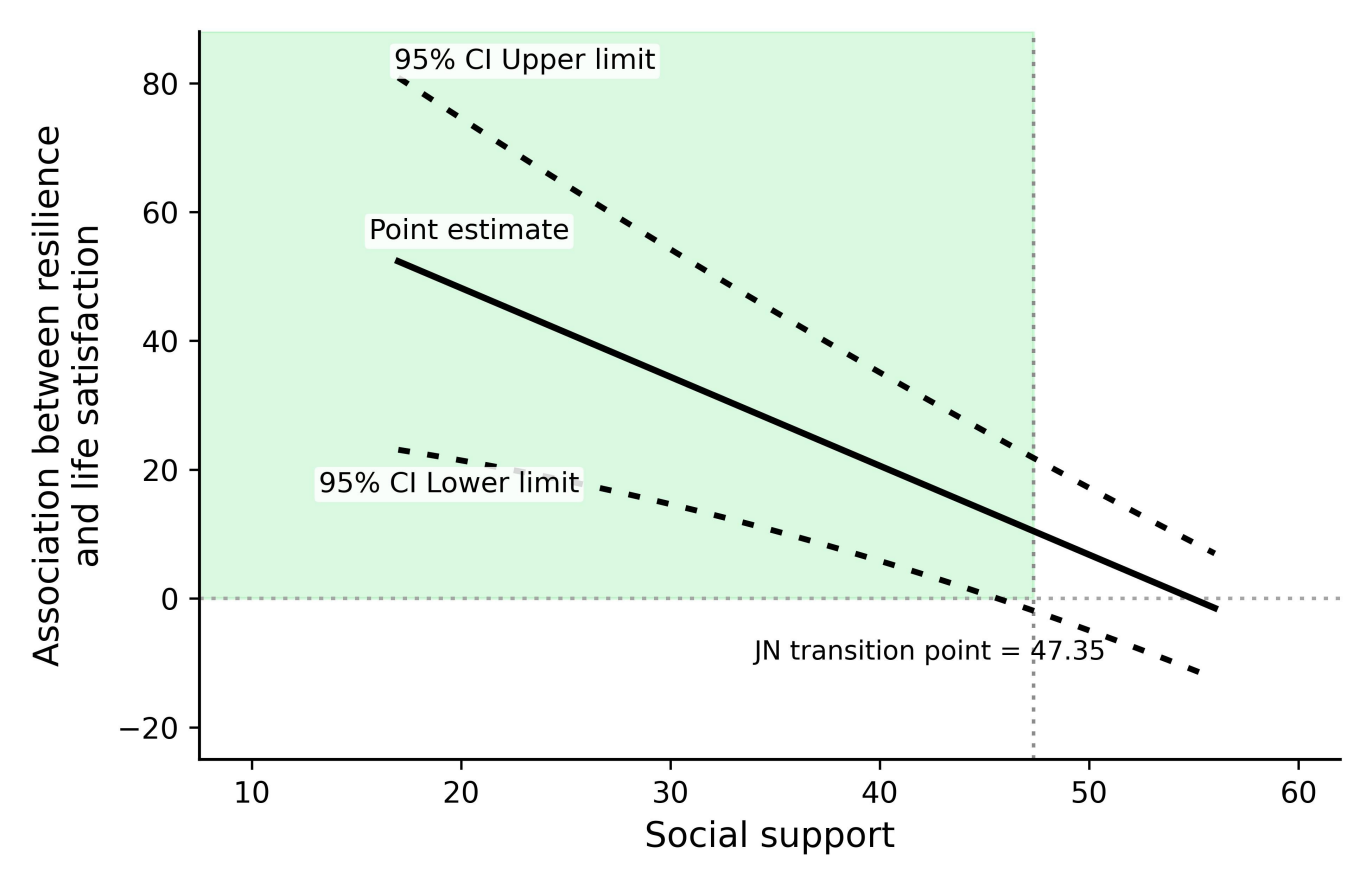
**Johnson-Neyman graph**. This graph illustrates the conditional association between resilience and life satisfaction as a linear function of social support. The plot includes the Johnson-Neyman transition point, which marks the value of social support at which the effect of resilience on life satisfaction becomes statistically significant. This point corresponds to where the CI for the conditional effect intersects zero on the Y-axis. The green shaded area represents the region of significance—that is, the range of social support values for which the association between resilience and life satisfaction is significant. CI, confidence interval; JN, Johnson-Neyman.

The Johnson-Neyman technique was utilized to pinpoint where social support levels shift the influence of resilience on life satisfaction. The results indicated that the effectiveness of resilience in enhancing life satisfaction becomes notably significant only when social support scores fall below 47.35 (see Fig. 1). At this Johnson-Neyman transition point, the conditional effect of resilience on life satisfaction was B = 0.0996, 95% CI (0.0000, 0.1993), indicating that this value represents the point at which the CI reaches zero. This conditional threshold was estimated using Hayes’ PROCESS macro (Model 1) and is expressed in the original raw-score metric of the perceived social support scale, as variables were not standardized prior to analysis. Given that the Duke-UNC-11 total score ranges from 11 to 55, and that scores below 32 are typically considered indicative of low perceived social support, this threshold suggests that the effect of resilience remained significant across a broad range of support levels and became non-significant only at relatively high levels of perceived social support. This threshold highlights a distinct divergence from the simpler effects modelled in the multiple regression, where the influence of individual predictors was not conditioned on the level of social support. In regions where social support exceeds this critical value, the relationship between resilience and life satisfaction does not manifest significantly. Conversely, with lower social support, this relationship not only emerges but also strengthens, underscoring the pivotal role of social environments in leveraging personal strengths like resilience.

In summary, the moderation analysis reveals that the impact of resilience on life satisfaction in a sample of healthy older people is contingent upon the level of social support, an insight that extends beyond the direct effects observed in the initial regression models. This nuanced understanding suggests that enhancing social support could be a key strategy in maximizing the positive effects of resilience on life satisfaction.

## Discussion

The main objective of this study was to analyze the relationship between resilience, perceived social support, and life satisfaction in healthy older adults. The overall results indicate that both resilience and the perceived social support positively and significantly predict their life satisfaction, one of the most studied indicators of subjective well-being in the scientific literature. Additionally, the results show that perceived social support moderates the relationship between psychological resilience and life satisfaction, with resilience becoming a stronger predictor when perceived social support is lower. Specifically, among individuals reporting higher levels of social support, the predictive contribution of resilience was reduced, whereas this association became stronger as perceived support decreased. These findings highlight the crucial role of an adequate social support network in fostering life satisfaction in late-life and emphasize how important resilience becomes when social support is lacking.

Based on the descriptive statistics of the study, participants perceive themselves as highly resilient, with good social support and a high level of life satisfaction. It is also noteworthy that these results are consistent across both men and women participants, with no significant gender differences found in the variables assessed. These results are in line with previous studies that have reported that aging adults generally indicate moderate to high levels of resilience [21, 26, 52], perceived medium to high social support [26, 30], and high life satisfaction [9, 26]. Moreover, several systematic reviews have emphasized that no gender differences are typically found in life satisfaction among this population [53], and that gender differences in resilience perception are either minimal or inconsistent [29]. Regarding social support, previous research has found no gender differences in the perceived social support among older adults, although gender differences were found in perceived instrumental support [54]. While these results depict a relatively positive situation for people in later life, it is important to point out that most studies, including the present one, have involved healthy, community-dwelling older adults, so differences could be expected if the study were conducted with individuals experiencing aging with serious illnesses.

Regarding the proposed hypotheses in the present study, the first one suggested that resilience and perceived social support will positively predict life satisfaction. The results confirm these relationships and indicate that when aging individuals feel resilient and capable of coping with adversity, this leads to greater life satisfaction. Studies involving different age groups suggest that those with higher resilience also report greater life satisfaction, possibly due to the personal resources they possess for coping with challenges [24, 55]. Specifically, evidence suggests that resilience contributes holistically to healthy aging and is positively related to life satisfaction and other well-being indicators such as quality of life, optimism or positive emotions [21, 26, 29]. On the other hand, the results of the present study show that when they perceive themselves as having a strong social support network—comprised of people they trust and who offer them affection and care—they experience greater life satisfaction. These findings align with previous research showing that social support is associated with higher life satisfaction, both in older adults [26, 30] and in other populations [35]. It is important to note that the absence of a significant association between resilience and perceived social support in the present study contrasts with some previous evidence. However, this result is not entirely inconsistent with the literature, as prior research has reported heterogeneous and context-dependent associations between these variables, including indirect or mediated relationships rather than direct correlations [26, 29]. This finding may be partly explained by the relatively homogeneous and high-functioning nature of the sample, as well as the limited sample size, which may have reduced the ability to detect smaller effects. Even more, comparing the effect of both independent variables on life satisfaction, the results of this study indicate that perceived social support has a greater impact on life satisfaction than resilience. Although previous literature does not show a consistent trend in these relationships [26, 39], these results underscore the importance of providing resources that help late-life people build resilience and strengthen their social support networks, as this contributes to greater well-being.

Regarding the second hypothesis, it was proposed that perceived social support could act as a moderator in the relationship between resilience and life satisfaction. The present results support this hypothesis, showing that the direct effect of resilience and the moderating role of social support together explain 44.98% of the variance in life satisfaction among older adults. This proportion of explained variance is higher than that reported in previous studies examining similar psychosocial predictors of life satisfaction in older adults [26]. Importantly, the inclusion of the interaction term contributed an additional 13.87% of explained variance in our study, suggesting a meaningful incremental contribution of the moderation model beyond main effects alone.

Furthermore, the analysis revealed that the impact of resilience on life satisfaction varies depending on the level of perceived social support. Specifically, as perceived social support increases, the predictive value of resilience decreases. This interaction effect suggests that the contribution of resilience to life satisfaction may depend on the availability of external psychosocial resources. This pattern is further clarified by the Johnson-Neyman analysis. An interesting aspect of this moderation finding is that the Johnson-Neyman threshold (47.35) lies well above the conventional Duke-UNC-11 cutoff used to identify low perceived social support (32 points). This indicates that the positive role of resilience in life satisfaction is not limited to individuals with clearly low social support, but extends across a broad range of support levels, becoming non-significant only when perceived support is especially high. This effect may indicate that resilience becomes particularly relevant when older adults cannot rely on exceptionally strong social support, whereas in highly supportive environments its unique contribution to life satisfaction is attenuated. In addition, social support may facilitate emotion regulation processes by buffering negative affect and stress responses, thereby enhancing life satisfaction even among individuals with lower levels of psychological resilience [56]. One possible explanation is a compensatory mechanism, whereby external resources such as social support may partially offset lower levels of individual resilience. This interpretation is supported by studies indicating that social support can play a compensatory role among individuals with lower resilience [57], as well as by broader theoretical and empirical work suggesting that psychosocial resources may operate in a buffering or non-additive manner [41, 58]. From a theoretical perspective, these findings can be understood within resource-based frameworks, particularly the Conservation of Resources theory [40], which proposes that psychosocial resources tend to cluster and may operate in interrelated and non-independent ways. In this sense, internal (e.g., resilience) and external (e.g., social support) resources may partially compensate for one another and jointly contribute to well-being, rather than exerting purely additive effects. This explanation is consistent with empirical evidence showing that social support can play a compensatory role among individuals with lower resilience [57], as well as with studies indicating that both resources interact in shaping well-being outcomes [41, 58]. This context-dependent pattern also aligns with the theoretical perspective outlined in the introduction, which proposed that psychosocial resources may operate in a compensatory and interactive manner. Overall, these findings suggest a context-dependent role of resilience, with greater relevance under conditions of limited social support. Importantly, the current results help clarify inconsistent evidence in the literature by supporting a moderation-based framework, suggesting that resilience and social support operate as interacting, rather than sequential, resources in shaping life satisfaction. Additionally, they provide empirical support for this model in healthy, community-dwelling older adults, a relatively understudied population in this context.

Along the same line, previous studies already found that social support exerted an important role in the relationship between resilience and variables such as quality of life [36], caregiver burnout [39], or sleep quality [59]. These studies support the idea that social support can be conceived as a protective factor for older adults [60]. Research conducted in other populations has also demonstrated associations between resilience, perceived social support, and subjective well-being [55], as well as with distress-related outcomes such as anxiety or depression in clinical populations [61]. It is important to note that some recent studies have approached these variables from a different perspective, finding that resilience partially mediated the relationship between social support and life satisfaction in older adults [26]. This mediation-based approach may appear to contrast with the moderation effect observed in the present study. However, these differences may be explained by several methodological and contextual factors. For instance, differences in sample characteristics may play a relevant role. Zheng* et al*. [26] examined a broader and more heterogeneous sample of older adults in a different cultural context, whereas the present study focused on a relatively homogeneous group of healthy, community-dwelling older adults with high levels of functioning. Such differences may influence the way in which internal and external resources interact. Moreover, the measurement and operationalization of variables may contribute to these discrepancies. In this sense, variations in the instruments used to assess resilience, social support or life satisfaction, as well as differences in how these constructs are conceptualized (e.g., perceived vs. received support), may lead to distinct patterns of association. And finally, mediation and moderation models capture different underlying processes; these approaches are not necessarily contradictory but may reflect complementary mechanisms operating simultaneously or under different conditions.

As the world’s population ages, the psychological and physical health of older adults has become a major global concern. The results of this study have important implications for promoting well-being in later life by identifying two key aspects that can foster life satisfaction: on one hand, the capacity for resilience to overcome adversity and grow through critical situations, and on the other hand the social support provided by family, friends, or professionals who care for them and show them affection. Importantly, the moderation results indicate that the effectiveness of resilience and social support may depend on their relative availability, which has direct implications for intervention design. Specifically, for older adults with low levels of perceived social support, interventions may benefit from prioritizing the development of individual resilience, such as coping strategies, emotional regulation or problem-solving skills. In contrast, when individuals report moderate or high levels of social support, interventions should focus on maintaining and optimizing existing social networks (e.g., enhancing relationship quality and promoting social participation). Therefore, the present research supports the need for tailored and multicomponent interventions, in which the balance between resilience training and social support enhancement is adjusted according to the participants’ psychosocial context. Previous research has shown that both types of interventions—those aimed at strengthening social support networks [37] and those focused on enhancing resilience [62]—can be effective in improving well-being in older adults.

Despite the contributions of this study, it is important to acknowledge some limitations. First, the sample size was modest, as it consisted of 42 healthy older adults, 71.4% of whom were women. Although a sensitivity analysis indicated that this sample was sufficient to detect moderate-to-large effects, the limited number of participants reduces statistical precision and constrains the generalizability of the findings. This issue is especially relevant for the moderation analysis, as interaction effects are often less stable than main effects in small samples and may be more sensitive to sampling variability. Second, the predominance of women in the sample may have influenced the estimation of the observed associations and limits the extent to which the results can be generalized to men. Therefore, the observed moderation pattern should be interpreted with caution until it is replicated in larger and more balanced samples. However, the gender difference observed in this study may reflect the composition of the specific sample, which consisted of individuals enrolled in a university course in the field of Psychology—a field predominantly chosen by women in the country of study. Moreover, previous studies have shown that women participate in these types of studies much more frequently than men in this stage of life [37, 63]. Third, participants were recruited from a university program for older people attending health-related courses, which means that the sample likely had better health and life conditions than their peers. Participants from this context are likely to represent a relatively active, healthy, and socially engaged subgroup, with potentially higher educational and psychosocial resources than general older population. Consequently, the results should be generalized with caution and primarily interpreted within the context of this specific participant profile. A similar situation has been observed in previous studies that also recruit healthy, community-dwelling older adults, where most participants tend to be in a positive situation in terms of health or social conditions [63]. Fourth, some potentially relevant contextual variables, such as economic level and social participation, were not available in the present dataset and therefore could not be considered in the analyses. This should be considered when interpreting the observed associations, as these factors may also influence perceived social support and life satisfaction. Finally, the cross-sectional design of the study precludes any causal interpretation of the observed relationships. Although the proposed model is theoretically grounded, longitudinal and experimental designs would be necessary to examine how these relationships evolve over time and to better establish causal pathways.

Future research would benefit from expanding the sample to include a larger number of participants, representative of both genders, and incorporating individuals from different social backgrounds and diverse health and social conditions, including more vulnerable populations such as frail older adults, those living in institutional settings and/or those with chronic health conditions, as previous research suggests that access to psychosocial resources and coping strategies may vary depending on health status and social context [64, 65]. Incorporating a broader range of contextual variables would also contribute to a more comprehensive understanding of the factors influencing life satisfaction in later life. In addition, further work should investigate the mechanisms underlying the observed moderation effect. The inclusion of longitudinal and experimental designs would be useful to clarify whether the interaction between resilience and social support reflects compensatory or non-additive processes, as suggested by previous research indicating that social support may play a compensatory role, particularly among individuals with lower psychological resilience [57], and that both resources may jointly influence well-being through direct and indirect pathways [41, 58]. Additionally, future research could include objective measures to strengthen the findings. While the instruments used are validated and have high experimental utility [26, 36, 66], the use of objective or multimethod approaches (e.g., behavioral, physiological, or informant-based measures) could strengthen the robustness of the findings.

## Conclusions

This study highlights two major promoters of life satisfaction in older adults: resilience and social support. These findings reinforce the importance of both individual psychological resources and social-contextual factors in promoting well-being during aging. Beyond replicating previous evidence on the positive role of these variables, the present study makes a theoretical contribution by advancing the understanding of how resilience and social support jointly operate in healthy older adults. Specifically, the results support a context-dependent and interactional perspective, showing that the effect of resilience on life satisfaction varies as a function of perceived social support. In this sense, the findings contribute to clarifying the ongoing debate in the literature regarding the interplay between these variables, providing empirical support for a moderation-based framework in a non-clinical aging population.

However, the scope of these findings should be interpreted considering the characteristics of the study sample. Participants were healthy, community-dwelling older adults recruited from a university-based program, which likely represents a relatively socially engaged and higher-functioning subgroup of the older population. This context may introduce a degree of selection bias, limiting the generalizability of the results to more vulnerable groups, such as older adults with poorer health status, lower educational levels, or reduced access to social resources.

From an applied perspective, the results suggest that promoting life satisfaction in older adults requires not only strengthening resilience but also improving social support networks. And importantly, the interaction observed between these variables indicates that interventions for this population may benefit from a multicomponent and personalized approach, considering the balance between their internal and external resources. Overall, this study contributes to a more integrative understanding of well-being in later life and provides a foundation for future research aimed at examining the dynamic of psychosocial resources across different aging contexts.

## Availability of Data and Materials

The data supporting the findings of this study are available upon reasonable request from the corresponding author.

## References

[1] Wang S (2020). Spatial patterns and social-economic influential factors of population aging: A global assessment from 1990 to 2010. *Social science & medicine*.

[2] World Health Organization (2024). Envejecimientoy Salud. https://www.who.int/es/news-room/fact-sheets/detail/ageing-and-health.

[3] Krivanek TJ, Gale SA, McFeeley BM, Nicastri CM, Daffner KR (2021). Promoting Successful Cognitive Aging: A Ten-Year Update. *Journal of Alzheimer’s Disease*.

[4] Holt-Lunstad J (2022). Social Connection as a Public Health Issue: the Evidence and a Systemic Framework for Prioritizing the “Social” in Social Determinants of Health. *Annual Review of Public Health*.

[5] Taylor MG, Carr D (2021). Psychological Resilience and Health Among Older Adults: A Comparison of Personal Resources. *The journals of gerontology: Series B, Psychological sciences and social sciences*.

[6] Santini ZI, Jose PE, York Cornwell E, Koyanagi A, Nielsen L, Hinrichsen C (2020). Social disconnectedness, perceived isolation, and symptoms of depression and anxiety among older Americans (NSHAP): A longitudinal mediation analysis. *The Lancet. Public health*.

[7] Livingston G, Huntley J, Sommerlad A, Ames D, Ballard C, Banerjee S (2020). Dementia prevention, intervention, and care: 2020 report of the Lancet Commission. *Lancet (London, England)*.

[8] Casamitjana M, Cuevas-Esteban J (2023). El envejecimiento saludable: Un desafío para la sociedad actual. *Neurosciences*.

[9] Tian H, Chen J (2022). Study on Life Satisfaction of the Elderly Based on Healthy Aging. *Journal of healthcare engineering*.

[10] Diener E, Emmons RA, Larsen RJ, Griffin S (1985). The Satisfaction With Life Scale. *Journal of personality assessment*.

[11] Kjell ONE, Daukantaitė D, Heferon K, Sikström S (2016). The harmony in life scale complements the satisfaction with life scale: Expanding the conceptualization of the cognitive component of subjective well-being. *Social Indicators Research*.

[12] Galambos NL (2020). The U Shape of Happiness across the Life Course: Expanding the Discussion. *Perspectives on Psychological Science*.

[13] An HY, Chen W, Wang CW, Yang HF, Huang WT, Fan SY (2020). The relationships between physical activity and life satisfaction and happiness among Young, middle-Aged, and older Adults. *International journal of environmental research and public health*.

[14] Rosella LC, Fu L, Buajitti E, Goel V (2019). Death and Chronic Disease Risk Associated With Poor Life Satisfaction: A Population-Based Cohort Study. *American journal of epidemiology*.

[15] Sone D, Beheshti I, Shinagawa S, Niimura H, Kobayashi N, Kida H (2022). Neuroimaging-derived brain age is associated with life satisfaction in cognitively unimpaired elderly: A community-based study. *Translational psychiatry*.

[16] Trică A, Golu F, Sava NI, Licu M, Zanfirescu ȘA, Adam R (2024). Resilience and successful aging: A systematic review and meta-analysis. *Acta psychologica*.

[17] Chen E, Jiang T, Chen MA, Miller GE (2024). Reflections on resilience. *Development and psychopathology*.

[18] Troy AS, Willroth EC, Shallcross AJ, Giuliani NR, Gross JJ, Mauss IB (2023). Psychological Resilience: An Affect-Regulation Framework. *Annual review of psychology*.

[19] Bartley EJ, Ofri BL, Vasilopoulos T, Palit S, Torres CA, Sibille KT (2024). Promoting a foundation of resilience in older adults: pilot trial of a strengths-based positive psychology intervention for chronic low back pain. *Health psychology and behavioral medicine*.

[20] Harvanek ZM, Fogelman N, Xu K, Sinha R (2021). Psychological and biological resilience modulates the effects of stress on epigenetic aging. *Translational psychiatry*.

[21] Zeng H, Liu Y, Zhang C, Zhang X, Shen M, Zhang Z (2024). The mediating effect of expectations regarding aging between psychological resilience and quality of life in rural elderly. *Archives of public health*.

[22] Chan SM, Chung GK, Chan YH, Chung RY, Wong H, Yeoh EK (2022). Resilience and coping strategies of older adults in Hong Kong during COVID-19 pandemic: a mixed methods study. *BMC geriatrics*.

[23] Gijzel SMW, Whitson HE, van de Leemput IA, Scheffer M, van Asselt D, Rector JL (2019). Resilience in Clinical Care: Getting a Grip on the Recovery Potential of Older Adults. *Journal of the American Geriatrics Society*.

[24] Guo Y (2017). Relationship between social support and life satisfaction of college students: resilience as a mediator and moderator. *Ethics in Progress*.

[25] Shabani M, Taheri-Kharameh Z, Saghafipour A, Ahmari-Tehran H, Yoosefee S, Amini-Tehrani M (2023). Resilience and spirituality mediate anxiety and life satisfaction in chronically Ill older adults. *BMC psychology*.

[26] Zheng W, Huang Y, Fu Y (2020). Mediating effects of psychological resilience on life satisfaction among older adults: a cross-sectional study in China. *Health & social care in the community*.

[27] Liao Z, Zhou H, He Z (2022). The mediating role of psychological resilience between social participation and life satisfaction among older adults in China. *BMC geriatrics*.

[28] Hiebel N, Rabe M, Maus K, Peusquens F, Radbruch L, Geiser F (2021). Resilience in Adult Health Science Revisited-A Narrative Review Synthesis of Process-Oriented Approaches. *Frontiers in psychology*.

[29] Górska S, Singh Roy A, Whitehall L, Irvine Fitzpatrick L, Duffy N, Forsyth K (2022). A Systematic Review and Correlational Meta-Analysis of Factors Associated With Resilience of Normally Aging, Community-Living Older Adults. *The Gerontologist*.

[30] Park S, Sok SR (2020). Relation Modeling of Factors Influencing Life Satisfaction and Adaptation of Korean Older Adults in Long-Term Care Facilities. *International journal of environmental research and public health*.

[31] Khodabakhsh S (2021). Factors Affecting Life Satisfaction of Older Adults in Asia: A Systematic Review. *Journal of Happiness Studies*.

[32] Li C, Jiang S, Li N, Zhang Q (2018). Influence of social participation on life satisfaction and depression among Chinese elderly: Social support as a mediator. *Journal of Community Psychology*.

[33] Cohen S, McKay G, Taylor SE, Singer JE, Baum A (2020). Social support, stress and the buffering hypothesis: a theoretical analysis. *Handbook of Psychology and Health IV*.

[34] Acoba EF (2024). Social support and mental health: the mediating role of perceived stress. *Frontiers in psychology*.

[35] Cao Q, Zhou Y (2021). Association between social support and life satisfaction among people with substance use disorder: the mediating role of resilience. *Journal of ethnicity in substance abuse*.

[36] Zhou K, Ning F, Wang X, Wang W, Han D, Li X (2022). Perceived social support and coping style as mediators between resilience and health-related quality of life in women newly diagnosed with breast cancer: a cross-sectional study. *BMC women’s health*.

[37] Czaja SJ, Boot WR, Charness N, Rogers WA, Sharit J (2018). Improving Social Support for Older Adults through Technology: Findings from the PRISM Randomized Controlled Trial. *The Gerontologist*.

[38] Liddle J, Stowell M, Ali M, Warwick S, Thompson A, Brittain K (2024). Community-based physical and social activity for older adults with mild frailty: A rapid qualitative study of a collaborative intervention pilot. *BMC geriatrics*.

[39] Ong HL, Vaingankar JA, Abdin E, Sambasivam R, Fauziana R, Tan ME (2018). Resilience and burden in caregivers of older adults: Moderating and mediating effects of perceived social support. *BMC psychiatry*.

[40] Hobfoll SE, Halbesleben J, Neveu JP, Westman M (2018). Conservation of Resources in the Organizational Context: The Reality of Resources and their Consequences. *Annual Review of Organizational Psychology and Organizational Behavior*.

[41] Kong LN, Zhang N, Yuan C, Yu ZY, Yuan W, Zhang GL (2021). Relationship of social support and health-related quality of life among migrant older adults: The mediating role of psychological resilience. *Geriatric nursing (New York, N.Y.)*.

[42] Lobo A, Saz P, Marcos G, Día JL, de la Cámara C, Ventura T (1999). Revalidation and standardization of the cognition mini-exam (first Spanish version of the Mini-Mental Status Examination) in the general geriatric population. *Medicina clínica*.

[43] Folstein MF, Folstein SE, McHugh PR (1975). “Mini-mental state”. A practical method for grading the cognitive state of patients for the clinician. *Journal of psychiatric research*.

[44] Connor KM, Davidson JR (2003). Development of a new resilience scale: The Connor-Davidson resilience scale (CD-RISC). *Depression and anxiety*.

[45] Broche-Pérez Y, Rodríguez-Martín BC, Pérez-Santaella S, Alonso-Díaz G, Hernández-Carballo A, Blanco-Consuegra Y, Rodríguez-Martín BC, Molerio-Pérez O (2012). Escala de Resiliencia de Connor-Davidson (CD-RISC). *Validación de instrumentos psicológicos: Criterios básicos*.

[46] Soler Sánchez MI, Meseguer de Pedro M, García Izquierdo M (2016). Psychometric properties of the spanish version of the 10-item Connor-Davidson resilience scale (10-item CD-RISC) in a sample of workers. *Revista Latinoamericana de Psicología*.

[47] Broadhead WE, Gehlbach SH, de Gruy FV, Kaplan BH (1988). The Duke-UNC Functional Social Support Questionnaire. Measurement of social support in family medicine patients. *Medical care*.

[48] Bellón JA, Delgado A, De Dios Luna J, Lardelli P (1996). Validity and reliability of the Duke-UNC-11 questionnaire of functional social support. *Atencion primaria*.

[49] Cuéllar-Flores I, Dresch V (2012). Validación del Cuestionario de Apoyo Social Funcional Duke-UNC-11 en personas cuidadoras. *Revista iberoamericana de diagnóstico y evaluación psicológica*.

[50] Atienza FL, Pons D, Balaguer I, García-Merita M (2000). Propiedades psicométricas de la Escala de Satisfacción con la Vida en adolescentes. *Psicothema*.

[51] Ramírez Pérez M, Lee Maturana SL (2012). Factores asociados a la satisfacción vital en adultos mayores de 60 años. *Polis. Revista Latinoamericana*.

[52] Treichler EBH, Glorioso D, Lee EE, Wu TC, Tu XM, Daly R (2020). A pragmatic trial of a group intervention in senior housing communities to increase resilience. *International psychogeriatrics*.

[53] Cheng A (2022). A systematic review of the associations, mediators and moderators of life satisfaction, positive affect and happiness in near-centenarians and centenarians. *Aging & mental health*.

[54] Abu-Kaf S, Nakash O, Hayat T, Cohen M (2022). Social support and psychological distress among the Bedouin Arab Elderly in Israel: The moderating role of gender. *International journal of environmental research and public health*.

[55] Yang C, Zhou Y, Xia M (2020). How resilience promotes mental health of patients with DSM-5 substance use disorder? The mediation roles of positive affect, self-esteem, and perceived social support. *Frontiers in psychiatry*.

[56] Southwick SM, Sippel L, Krystal J, Charney D, Mayes L, Pietrzak R (2016). Why are some individuals more resilient than others: the role of social support. *World psychiatry : official journal of the World Psychiatric Association (WPA)*.

[57] Tadai ME, Straughan PT, Cheong G, Yi RNW, Er TY (2023). The effects of SES, social support, and resilience on older adults’ well-being during COVID-19: Evidence from Singapore. *Urban Governance*.

[58] Upasen R, Saengpanya W, Awae W, Prasitvej P (2024). The influence of resilience and social support on mental health of older adults living in community: a cross-sectional study. *BMC psychology*.

[59] Cui C, Wang L (2024). Role of social support in the relationship between resilience and sleep quality among cancer patients. *Frontiers in psychiatry*.

[60] Cihlar V, Micheel F, Mergenthaler A (2023). Multidimensional vulnerability among older adults in Germany: Social support buffers the negative association with life satisfaction. *Zeitschrift fur Gerontologie und Geriatrie*.

[61] Sippel LM, Pietrzak RH, Charney DS, Mayes LC, Southwick SM (2015). How does social support enhance resilience in the trauma-exposed individual?. *Ecology and Society*.

[62] Terkes N, Aksu NT, Yamac SU (2023). The effect of an online-supervised exercise program in older people with diabetes on fasting blood sugar, psychological resilience and quality of life: a double blind randomised controlled trial. *International journal of older people nursing*.

[63] Rae C, Byles J, Denholm S, Holford J, Chojenta C (2025). Connections for Ageing well: a community healthy ageing program to promote social connection. *Australasian journal on ageing*.

[64] Liao Z, Zhou H, He Z (2022). The mediating role of psychological resilience between social participation and life satisfaction among older adults in China. *BMC geriatrics*.

[65] Song H, Li Z (2023). Community-based service, psychological resilience and life satisfaction among Chinese older adults: A longitudinal study. *Geriatric nursing (New York, N.Y.)*.

[66] Ruiz-Comellas A, Sauch Valmaña G, Mendioroz Peña J, Roura Poch P, Sabata Carrera A, Cornet Pujol I (2021). Physical activity, emotional state and socialization in the elderly: study protocol for a clinical multicentre randomized trial. *The Journal of international medical research*.

